# Fahr’s Disease: a case report of a patient with neuropsychiatric symptoms

**DOI:** 10.1192/j.eurpsy.2023.1622

**Published:** 2023-07-19

**Authors:** I. Perez-Sagaseta, S. Yelmo-Cruz, C. Cardenes-Moreno, L. Torres-Tejera, A. Crisostomo-Siverio, J. Dorta-Gonzalez, J. J. Tascon-Cervera, M. Paniagua-Gonzalez, S. Canessa, M. R. Cejas-Mendez

**Affiliations:** Psychiatry, Hospital Universitario de Canarias, La Laguna, Spain

## Abstract

**Introduction:**

Fahr’s disease (FD) is a rare disorder consisting of bilateral and symmetrical calcium deposits in basal ganglia and cerebral cortex. These lesions are associated with neurological and psychiatric symptoms such as a rigid hypokinetic syndrome, mood disorders and memory and concentration abnormalities. It can be idiopathic or secondary to endocrine disorders, infectious diseases or mitochondrial myopathies.

**Objectives:**

To highlight the importance of considering organic causes when evaluating patients presenting atypical psychiatric symptoms and claim the role of neuroimaging.

**Methods:**

Case report and non-systematic review of literature: sources obtained from Pubmed database.

**Results:**

A 69-year-old man, native of Syracuse (Italy), was admitted to the Psychiatry Unit in February 2022 presenting behavioural disturbances and irritability. In July 2021 he presented the same symptoms, being mistakenly diagnosed with Bipolar Disease type I. He has no previous psychiatric history. He started with changes in his personality, short-term memory loss, aggressiveness and disorganized behaviour at the age of 66. At admission he was talkative and hyperfamiliar, presenting delusions of grandiosity, exalted affectivity and insomnia. Neurological examination showed short-term memory problems, signs of frontal disinhibition and abnormal glabellar tap sign. Blood tests, CT brain and MRI were performed to rule out organic underlying causes. Neuro-imaging found bilateral and symmetric calcifications in globus pallidus, thalamus and corpus striatum, in favour of FD. Secondary causes (abnormalities in the PTH, vitamin disorders and infectious diseases such as HIV, brucellosis or neurosyphilis) where discarded, allowing us to conclude it was probably a primary case of FD. Valproate was started as a mood stabilizer and anticonvulsant. Genetic tests were indicated.

**Conclusions:**

FD should be considered as a differential diagnosis in the evaluation of psychiatric symptoms, especially when atypical and/or presented with neurological symptoms. The role of neuro-imaging is essential.

**Disclosure of Interest:**

None Declared

